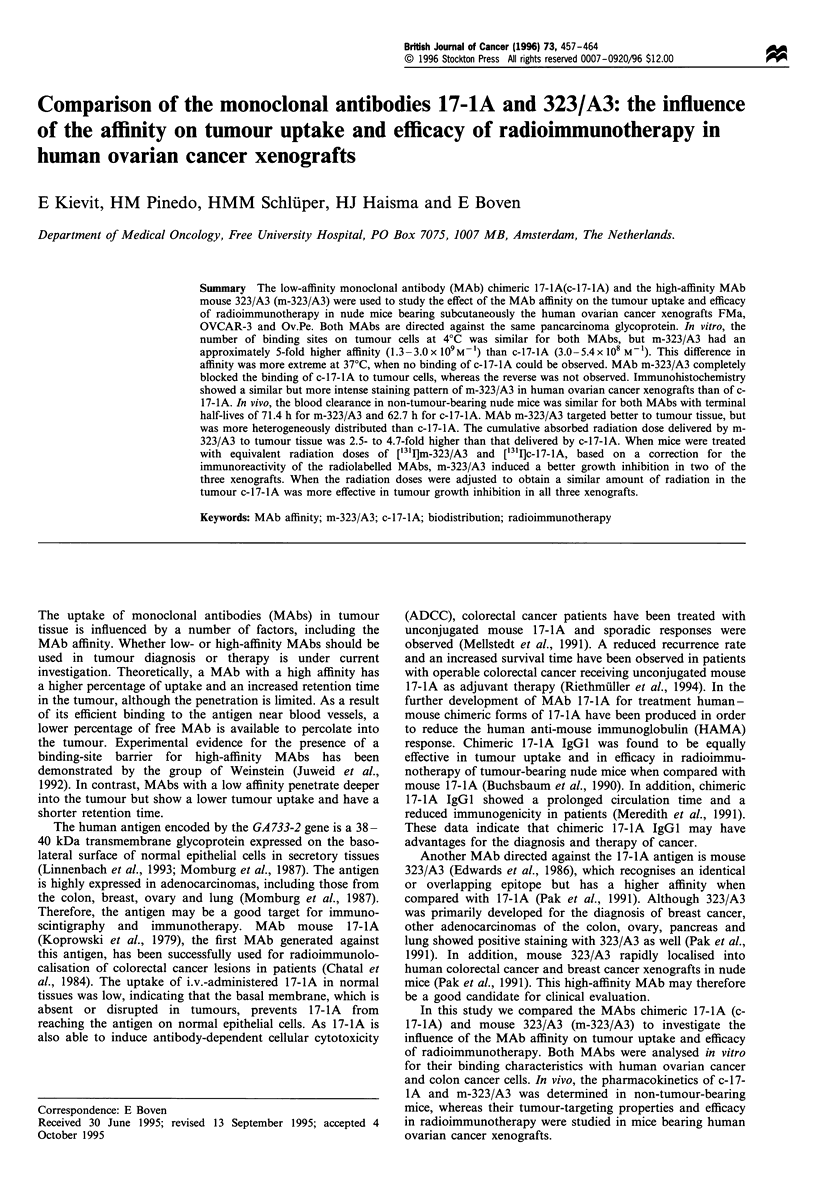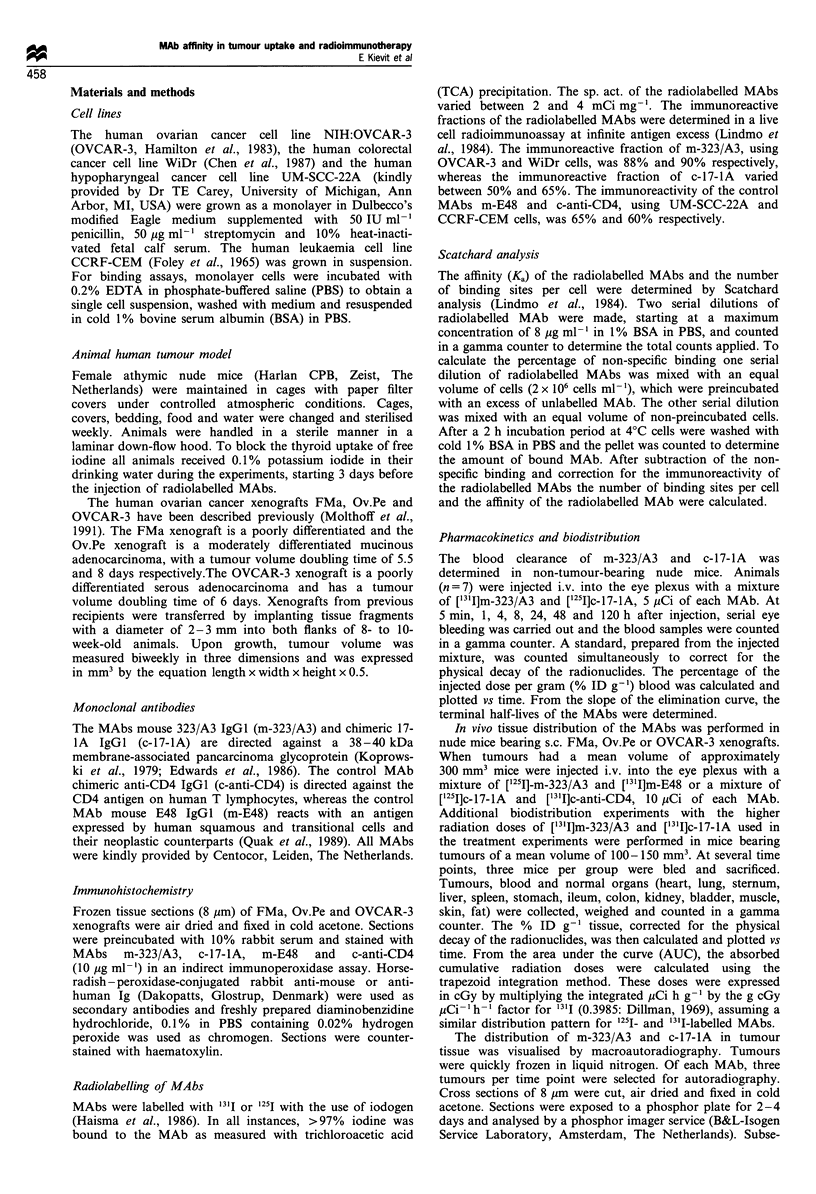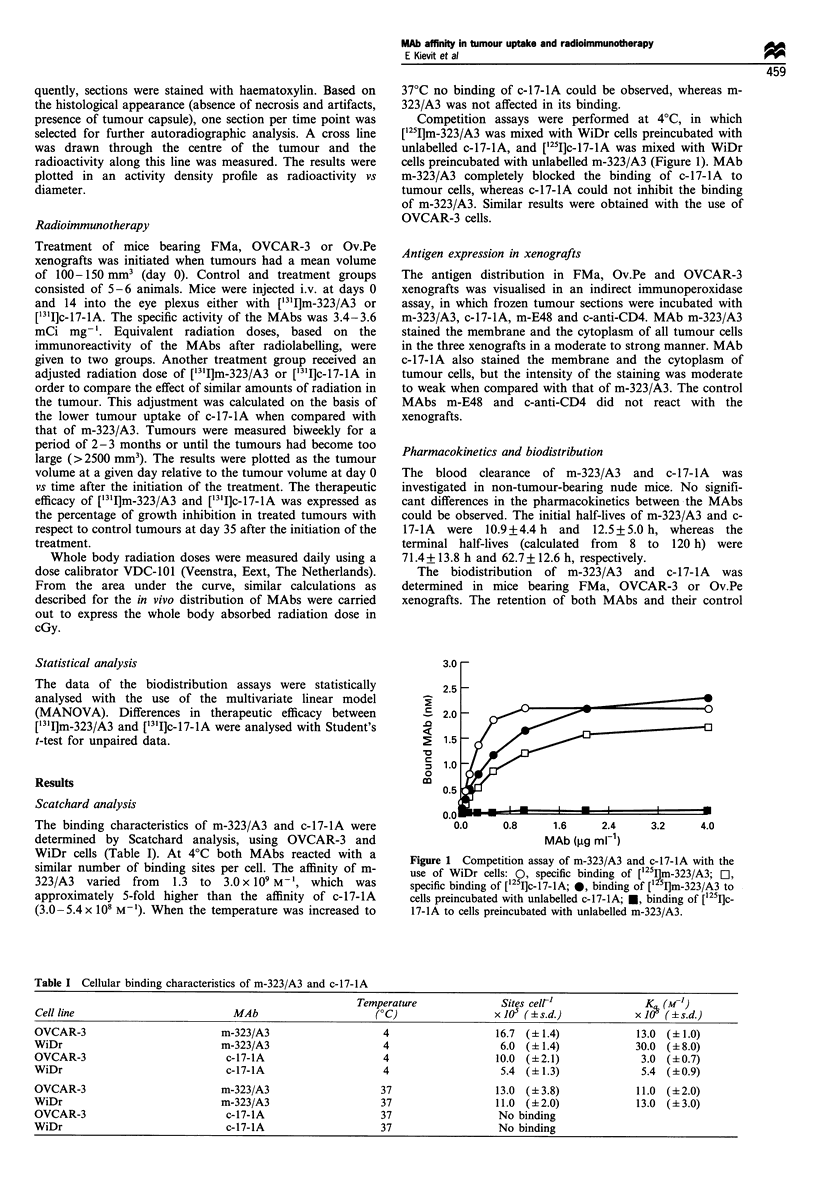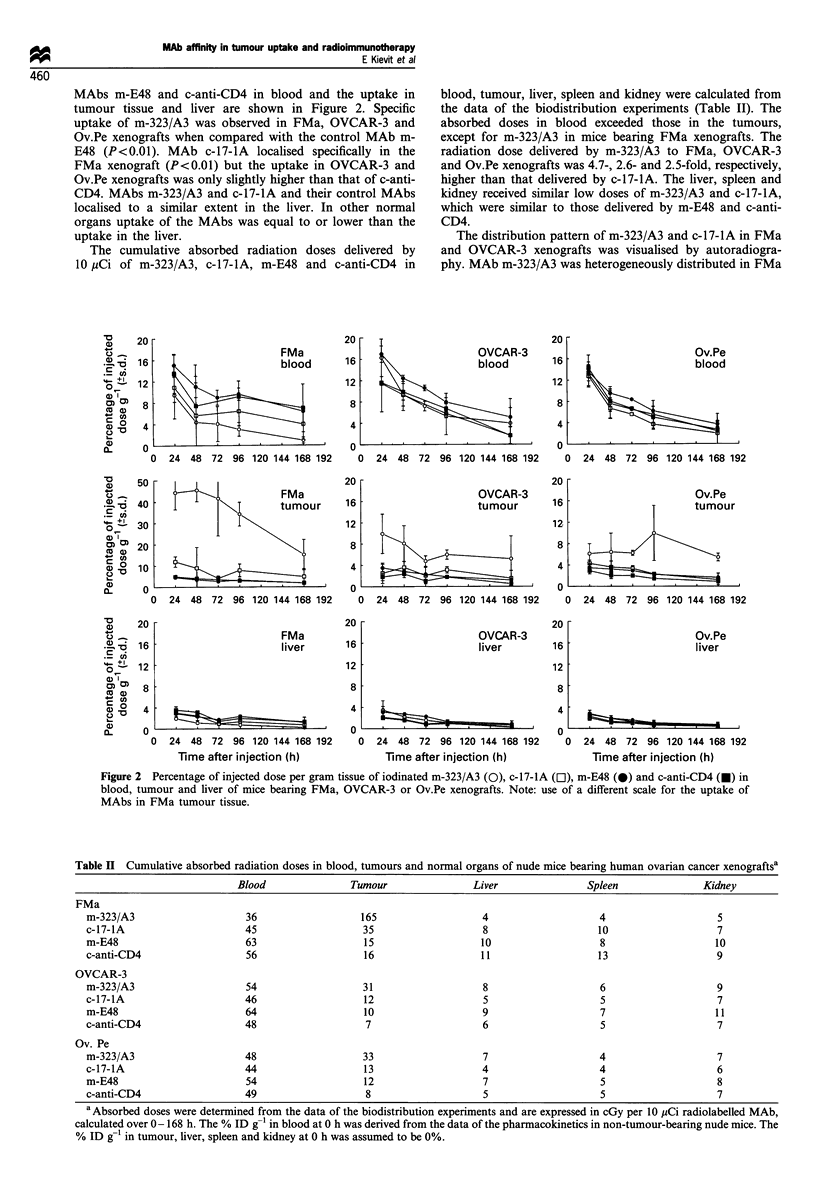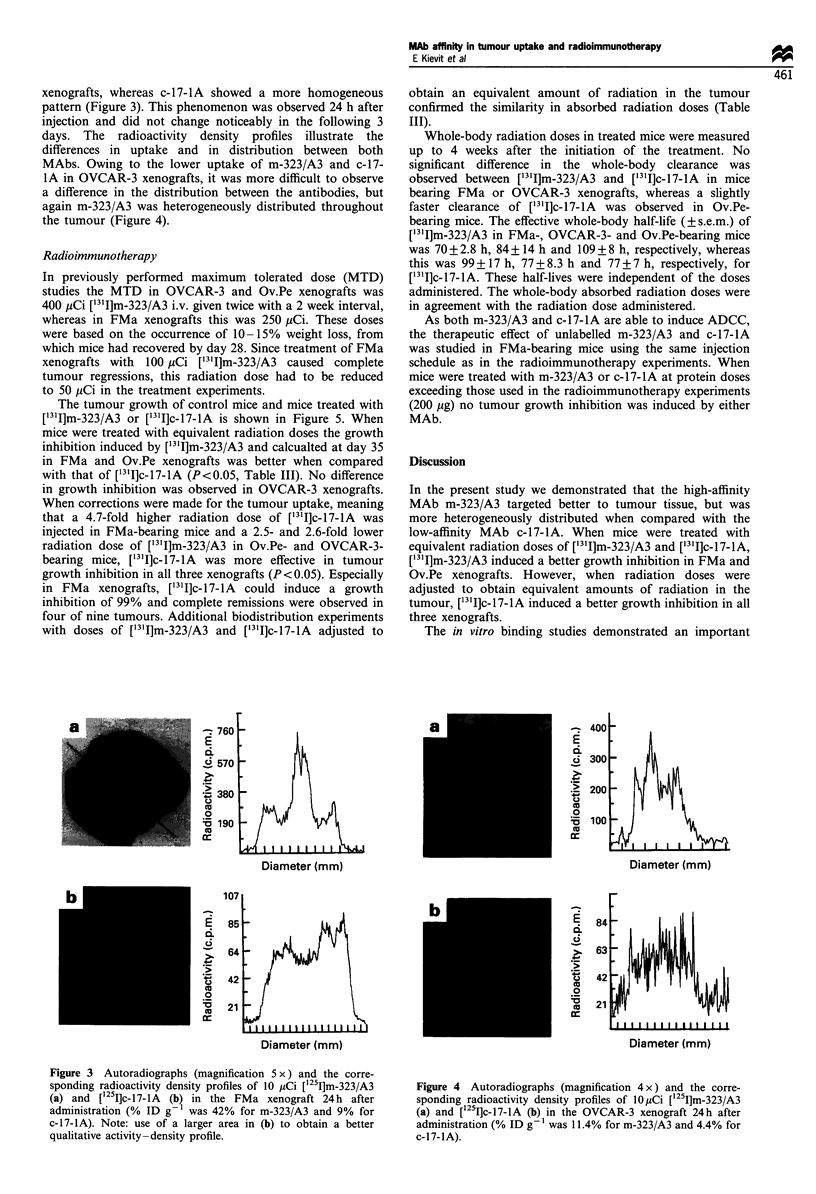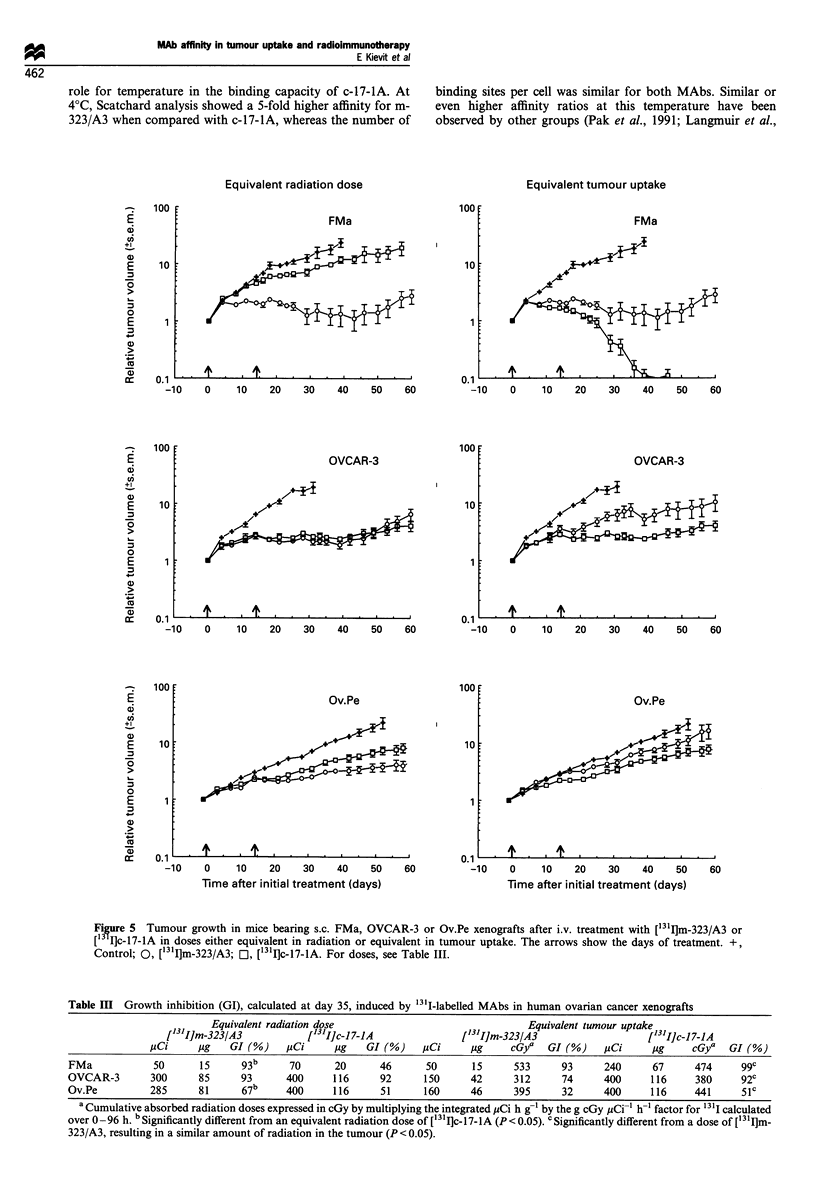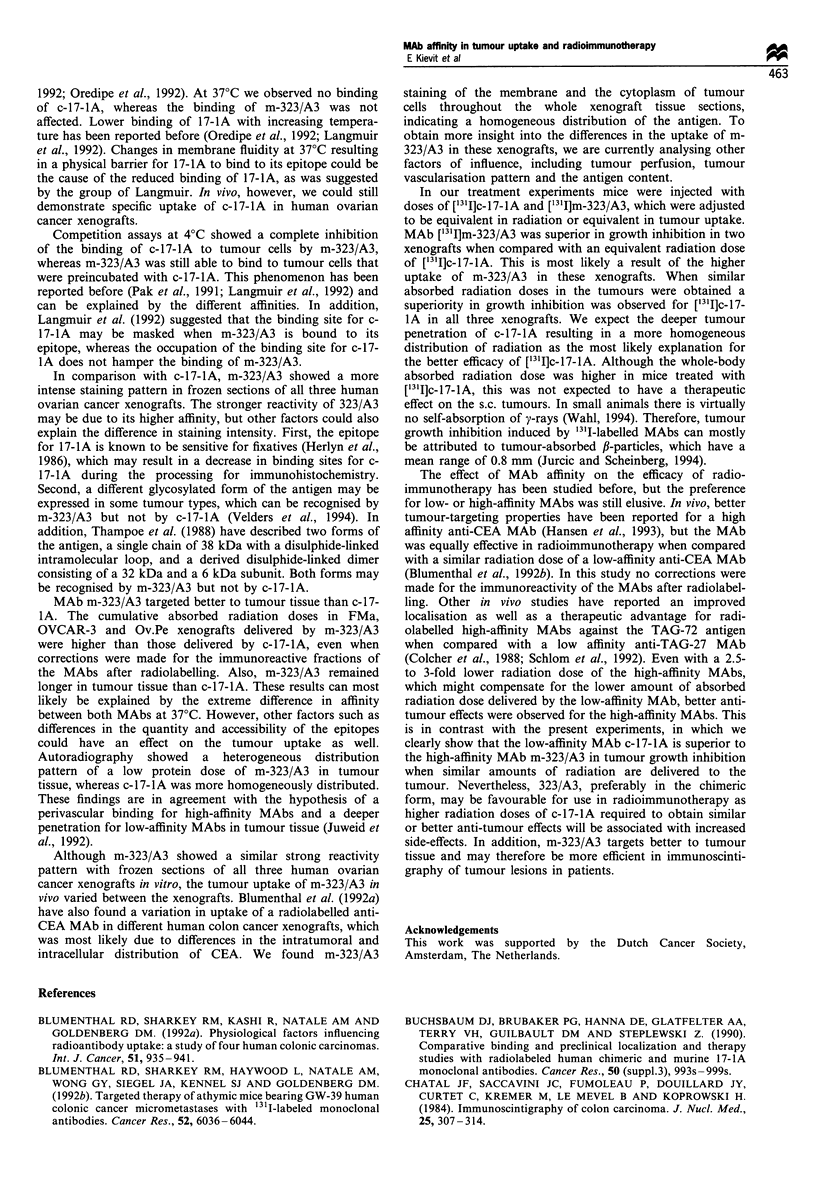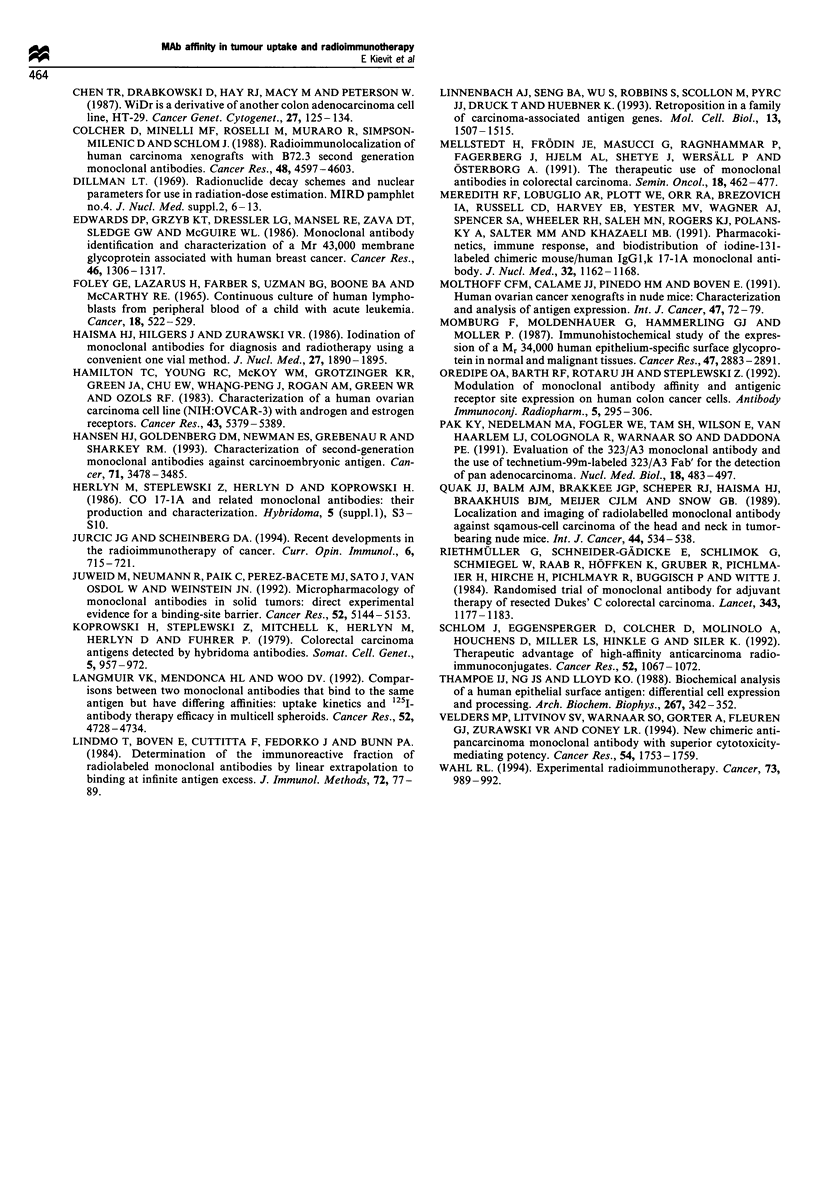# Comparison of monoclonal antibodies 17-1A and 323/A3: the influence of the affinity on tumour uptake and efficacy of radioimmunotherapy in human ovarian cancer xenografts.

**DOI:** 10.1038/bjc.1996.81

**Published:** 1996-02

**Authors:** E. Kievit, H. M. Pinedo, H. M. Schlüper, H. J. Haisma, E. Boven

**Affiliations:** Department of Medical Oncology, Free University Hospital, Amsterdam, The Netherlands.

## Abstract

**Images:**


British Journal of Cancer (1996) 73, 457-464
© 1996 Stockton Press All rights reserved 0007-0920/96 $12.00

### Comparison of the monoclonal antibodies 17-1A and 323/A3: the influence of the affinity on tumour uptake and efficacy of radioimmunotherapy in human ovarian cancer xenografts

E Kievit, HM Pinedo, HMM Schlüper, HJ Haisma and E Boven

*Department of Medical Oncology, Free University Hospital, PO Box 7075, 1007 MB, Amsterdam, The Netherlands.*

**Summary** The low-affinity monoclonal antibody (MAb) chimeric 17-1A(c-17-1A) and the high-affinity MAb mouse 323/A3 (m-323/A3) were used to study the effect of the MAb affinity on the tumour uptake and efficacy of radioimmunotherapy in nude mice bearing subcutaneously the human ovarian cancer xenografts FMa, OVCAR-3 and Ov.Pe. Both MAbs are directed against the same pancarcinoma glycoprotein. *In vitro*, the number of binding sites on tumour cells at 4°C was similar for both MAbs, but m-323/A3 had an approximately 5-fold higher affinity ($1.3-3.0 \times 10^9 \text{M}^{-1}$) than c-17-1A ($3.0-5.4 \times 10^8 \text{M}^{-1}$). This difference in affinity was more extreme at 37°C, when no binding of c-17-1A could be observed. MAb m-323/A3 completely blocked the binding of c-17-1A to tumour cells, whereas the reverse was not observed. Immunohistochemistry showed a similar but more intense staining pattern of m-323/A3 in human ovarian cancer xenografts than of c-17-1A. *In vivo*, the blood clearance in non-tumour-bearing nude mice was similar for both MAbs with terminal half-lives of 71.4 h for m-323/A3 and 62.7 h for c-17-1A. MAb m-323/A3 targeted better to tumour tissue, but was more heterogeneously distributed than c-17-1A. The cumulative absorbed radiation dose delivered by m-323/A3 to tumour tissue was 2.5- to 4.7-fold higher than that delivered by c-17-1A. When mice were treated with equivalent radiation doses of [$^{131}$I]m-323/A3 and [$^{131}$I]c-17-1A, based on a correction for the immunoreactivity of the radiolabelled MAbs, m-323/A3 induced a better growth inhibition in two of the three xenografts. When the radiation doses were adjusted to obtain a similar amount of radiation in the tumour c-17-1A was more effective in tumour growth inhibition in all three xenografts.

**Keywords:** MAb affinity; m-323/A3; c-17-1A; biodistribution; radioimmunotherapy

The uptake of monoclonal antibodies (MAbs) in tumour tissue is influenced by a number of factors, including the MAb affinity. Whether low- or high-affinity MAbs should be used in tumour diagnosis or therapy is under current investigation. Theoretically, a MAb with a high affinity has a higher percentage of uptake and an increased retention time in the tumour, although the penetration is limited. As a result of its efficient binding to the antigen near blood vessels, a lower percentage of free MAb is available to percolate into the tumour. Experimental evidence for the presence of a binding-site barrier for high-affinity MAbs has been demonstrated by the group of Weinstein (Juweid *et al.*, 1992). In contrast, MAbs with a low affinity penetrate deeper into the tumour but show a lower tumour uptake and have a shorter retention time.

The human antigen encoded by the *GA733-2* gene is a 38-40 kDa transmembrane glycoprotein expressed on the basolateral surface of normal epithelial cells in secretory tissues (Linnenbach *et al.*, 1993; Momburg *et al.*, 1987). The antigen is highly expressed in adenocarcinomas, including those from the colon, breast, ovary and lung (Momburg *et al.*, 1987). Therefore, the antigen may be a good target for immunoscintigraphy and immunotherapy. MAb mouse 17-1A (Koprowski *et al.*, 1979), the first MAb generated against this antigen, has been successfully used for radioimmunolocalisation of colorectal cancer lesions in patients (Chatal *et al.*, 1984). The uptake of i.v.-administered 17-1A in normal tissues was low, indicating that the basal membrane, which is absent or disrupted in tumours, prevents 17-1A from reaching the antigen on normal epithelial cells. As 17-1A is also able to induce antibody-dependent cellular cytotoxicity (ADCC), colorectal cancer patients have been treated with unconjugated mouse 17-1A and sporadic responses were observed (Mellstedt *et al.*, 1991). A reduced recurrence rate and an increased survival time have been observed in patients with operable colorectal cancer receiving unconjugated mouse 17-1A as adjuvant therapy (Riethmüller *et al.*, 1994). In the further development of MAb 17-1A for treatment human-mouse chimeric forms of 17-1A have been produced in order to reduce the human anti-mouse immunoglobulin (HAMA) response. Chimeric 17-1A IgG1 was found to be equally effective in tumour uptake and in efficacy in radioimmunotherapy of tumour-bearing nude mice when compared with mouse 17-1A (Buchsbaum *et al.*, 1990). In addition, chimeric 17-1A IgG1 showed a prolonged circulation time and a reduced immunogenicity in patients (Meredith *et al.*, 1991). These data indicate that chimeric 17-1A IgG1 may have advantages for the diagnosis and therapy of cancer.

Another MAb directed against the 17-1A antigen is mouse 323/A3 (Edwards *et al.*, 1986), which recognises an identical or overlapping epitope but has a higher affinity when compared with 17-1A (Pak *et al.*, 1991). Although 323/A3 was primarily developed for the diagnosis of breast cancer, other adenocarcinomas of the colon, ovary, pancreas and lung showed positive staining with 323/A3 as well (Pak *et al.*, 1991). In addition, mouse 323/A3 rapidly localised into human colorectal cancer and breast cancer xenografts in nude mice (Pak *et al.*, 1991). This high-affinity MAb may therefore be a good candidate for clinical evaluation.

In this study we compared the MAbs chimeric 17-1A (c-17-1A) and mouse 323/A3 (m-323/A3) to investigate the influence of the MAb affinity on tumour uptake and efficacy of radioimmunotherapy. Both MAbs were analysed *in vitro* for their binding characteristics with human ovarian cancer and colon cancer cells. *In vivo*, the pharmacokinetics of c-17-1A and m-323/A3 was determined in non-tumour-bearing mice, whereas their tumour-targeting properties and efficacy in radioimmunotherapy were studied in mice bearing human ovarian cancer xenografts.

Correspondence: E Boven
Received 30 June 1995; revised 13 September 1995; accepted 4 October 1995

### Materials and methods

### *Cell lines*

The human ovarian cancer cell line NIH:OVCAR-3 (OVCAR-3, Hamilton *et al.*, 1983), the human colorectal cancer cell line WiDr (Chen *et al.*, 1987) and the human hypopharyngeal cancer cell line UM-SCC-22A (kindly provided by Dr TE Carey, University of Michigan, Ann Arbor, MI, USA) were grown as a monolayer in Dulbecco's modified Eagle medium supplemented with 50 IU $ml^{-1}$ penicillin, 50 $\mu g\ ml^{-1}$ streptomycin and 10% heat-inactivated fetal calf serum. The human leukaemia cell line CCRF-CEM (Foley *et al.*, 1965) was grown in suspension. For binding assays, monolayer cells were incubated with 0.2% EDTA in phosphate-buffered saline (PBS) to obtain a single cell suspension, washed with medium and resuspended in cold 1% bovine serum albumin (BSA) in PBS.

### *Animal human tumour model*

Female athymic nude mice (Harlan CPB, Zeist, The Netherlands) were maintained in cages with paper filter covers under controlled atmospheric conditions. Cages, covers, bedding, food and water were changed and sterilised weekly. Animals were handled in a sterile manner in a laminar down-flow hood. To block the thyroid uptake of free iodine all animals received 0.1% potassium iodide in their drinking water during the experiments, starting 3 days before the injection of radiolabelled MAbs.

The human ovarian cancer xenografts FMa, Ov.Pe and OVCAR-3 have been described previously (Molthoff *et al.*, 1991). The FMa xenograft is a poorly differentiated and the Ov.Pe xenograft is a moderately differentiated mucinous adenocarcinoma, with a tumour volume doubling time of 5.5 and 8 days respectively.The OVCAR-3 xenograft is a poorly differentiated serous adenocarcinoma and has a tumour volume doubling time of 6 days. Xenografts from previous recipients were transferred by implanting tissue fragments with a diameter of 2–3 mm into both flanks of 8- to 10-week-old animals. Upon growth, tumour volume was measured biweekly in three dimensions and was expressed in $mm^3$ by the equation length × width × height × 0.5.

### *Monoclonal antibodies*

The MAbs mouse 323/A3 IgG1 (m-323/A3) and chimeric 17-1A IgG1 (c-17-1A) are directed against a 38–40 kDa membrane-associated pancarcinoma glycoprotein (Koprowski *et al.*, 1979; Edwards *et al.*, 1986). The control MAb chimeric anti-CD4 IgG1 (c-anti-CD4) is directed against the CD4 antigen on human T lymphocytes, whereas the control MAb mouse E48 IgG1 (m-E48) reacts with an antigen expressed by human squamous and transitional cells and their neoplastic counterparts (Quak *et al.*, 1989). All MAbs were kindly provided by Centocor, Leiden, The Netherlands.

### *Immunohistochemistry*

Frozen tissue sections (8 $\mu$m) of FMa, Ov.Pe and OVCAR-3 xenografts were air dried and fixed in cold acetone. Sections were preincubated with 10% rabbit serum and stained with MAbs m-323/A3, c-17-1A, m-E48 and c-anti-CD4 (10 $\mu g\ ml^{-1}$) in an indirect immunoperoxidase assay. Horse-radish–peroxidase-conjugated rabbit anti-mouse or anti-human Ig (Dakopatts, Glostrup, Denmark) were used as secondary antibodies and freshly prepared diaminobenzidine hydrochloride, 0.1% in PBS containing 0.02% hydrogen peroxide was used as chromogen. Sections were counter-stained with haematoxylin.

### *Radiolabelling of MAbs*

MAbs were labelled with $^{131}I$ or $^{125}I$ with the use of iodogen (Haisma *et al.*, 1986). In all instances, >97% iodine was bound to the MAb as measured with trichloroacetic acid (TCA) precipitation. The sp. act. of the radiolabelled MAbs varied between 2 and 4 mCi $mg^{-1}$. The immunoreactive fractions of the radiolabelled MAbs were determined in a live cell radioimmunoassay at infinite antigen excess (Lindmo *et al.*, 1984). The immunoreactive fraction of m-323/A3, using OVCAR-3 and WiDr cells, was 88% and 90% respectively, whereas the immunoreactive fraction of c-17-1A varied between 50% and 65%. The immunoreactivity of the control MAbs m-E48 and c-anti-CD4, using UM-SCC-22A and CCRF-CEM cells, was 65% and 60% respectively.

### *Scatchard analysis*

The affinity ($K_a$) of the radiolabelled MAbs and the number of binding sites per cell were determined by Scatchard analysis (Lindmo *et al.*, 1984). Two serial dilutions of radiolabelled MAb were made, starting at a maximum concentration of 8 $\mu g\ ml^{-1}$ in 1% BSA in PBS, and counted in a gamma counter to determine the total counts applied. To calculate the percentage of non-specific binding one serial dilution of radiolabelled MAbs was mixed with an equal volume of cells ($2 \times 10^6$ cells $ml^{-1}$), which were preincubated with an excess of unlabelled MAb. The other serial dilution was mixed with an equal volume of non-preincubated cells. After a 2 h incubation period at 4°C cells were washed with cold 1% BSA in PBS and the pellet was counted to determine the amount of bound MAb. After subtraction of the non-specific binding and correction for the immunoreactivity of the radiolabelled MAbs the number of binding sites per cell and the affinity of the radiolabelled MAb were calculated.

### *Pharmacokinetics and biodistribution*

The blood clearance of m-323/A3 and c-17-1A was determined in non-tumour-bearing nude mice. Animals ($n = 7$) were injected i.v. into the eye plexus with a mixture of [$^{131}I$]m-323/A3 and [$^{125}I$]c-17-1A, 5 $\mu$Ci of each MAb. At 5 min, 1, 4, 8, 24, 48 and 120 h after injection, serial eye bleeding was carried out and the blood samples were counted in a gamma counter. A standard, prepared from the injected mixture, was counted simultaneously to correct for the physical decay of the radionuclides. The percentage of the injected dose per gram (% ID $g^{-1}$) blood was calculated and plotted *vs* time. From the slope of the elimination curve, the terminal half-lives of the MAbs were determined.

*In vivo* tissue distribution of the MAbs was performed in nude mice bearing s.c. FMa, Ov.Pe or OVCAR-3 xenografts. When tumours had a mean volume of approximately 300 $mm^3$ mice were injected i.v. into the eye plexus with a mixture of [$^{125}I$]-m-323/A3 and [$^{131}I$]m-E48 or a mixture of [$^{125}I$]c-17-1A and [$^{131}I$]c-anti-CD4, 10 $\mu$Ci of each MAb. Additional biodistribution experiments with the higher radiation doses of [$^{131}I$]m-323/A3 and [$^{131}I$]c-17-1A used in the treatment experiments were performed in mice bearing tumours of a mean volume of 100–150 $mm^3$. At several time points, three mice per group were bled and sacrificed. Tumours, blood and normal organs (heart, lung, sternum, liver, spleen, stomach, ileum, colon, kidney, bladder, muscle, skin, fat) were collected, weighed and counted in a gamma counter. The % ID $g^{-1}$ tissue, corrected for the physical decay of the radionuclides, was then calculated and plotted *vs* time. From the area under the curve (AUC), the absorbed cumulative radiation doses were calculated using the trapezoid integration method. These doses were expressed in cGy by multiplying the integrated $\mu$Ci h $g^{-1}$ by the g cGy $\mu Ci^{-1}\ h^{-1}$ factor for $^{131}I$ (0.3985: Dillman, 1969), assuming a similar distribution pattern for $^{125}I$- and $^{131}I$-labelled MAbs.

The distribution of m-323/A3 and c-17-1A in tumour tissue was visualised by macroautoradiography. Tumours were quickly frozen in liquid nitrogen. Of each MAb, three tumours per time point were selected for autoradiography. Cross sections of 8 $\mu$m were cut, air dried and fixed in cold acetone. Sections were exposed to a phosphor plate for 2–4 days and analysed by a phosphor imager service (B&L-Isogen Service Laboratory, Amsterdam, The Netherlands). Subsequently, sections were stained with haematoxylin. Based on the histological appearance (absence of necrosis and artifacts, presence of tumour capsule), one section per time point was selected for further autoradiographic analysis. A cross line was drawn through the centre of the tumour and the radioactivity along this line was measured. The results were plotted in an activity density profile as radioactivity *vs* diameter.

*Radioimmunotherapy*

Treatment of mice bearing FMa, OVCAR-3 or Ov.Pe xenografts was initiated when tumours had a mean volume of 100–150 mm$^3$ (day 0). Control and treatment groups consisted of 5–6 animals. Mice were injected i.v. at days 0 and 14 into the eye plexus either with [$^{131}$I]m-323/A3 or [$^{131}$I]c-17-1A. The specific activity of the MAbs was 3.4–3.6 mCi mg$^{-1}$. Equivalent radiation doses, based on the immunoreactivity of the MAbs after radiolabelling, were given to two groups. Another treatment group received an adjusted radiation dose of [$^{131}$I]m-323/A3 or [$^{131}$I]c-17-1A in order to compare the effect of similar amounts of radiation in the tumour. This adjustment was calculated on the basis of the lower tumour uptake of c-17-1A when compared with that of m-323/A3. Tumours were measured biweekly for a period of 2–3 months or until the tumours had become too large (>2500 mm$^3$). The results were plotted as the tumour volume at a given day relative to the tumour volume at day 0 *vs* time after the initiation of the treatment. The therapeutic efficacy of [$^{131}$I]m-323/A3 and [$^{131}$I]c-17-1A was expressed as the percentage of growth inhibition in treated tumours with respect to control tumours at day 35 after the initiation of the treatment.

Whole body radiation doses were measured daily using a dose calibrator VDC-101 (Veenstra, Eext, The Netherlands). From the area under the curve, similar calculations as described for the *in vivo* distribution of MAbs were carried out to express the whole body absorbed radiation dose in cGy.

*Statistical analysis*

The data of the biodistribution assays were statistically analysed with the use of the multivariate linear model (MANOVA). Differences in therapeutic efficacy between [$^{131}$I]m-323/A3 and [$^{131}$I]c-17-1A were analysed with Student's *t*-test for unpaired data.

### Results

*Scatchard analysis*

The binding characteristics of m-323/A3 and c-17-1A were determined by Scatchard analysis, using OVCAR-3 and WiDr cells (Table I). At 4°C both MAbs reacted with a similar number of binding sites per cell. The affinity of m-323/A3 varied from 1.3 to $3.0 \times 10^9$ M$^{-1}$, which was approximately 5-fold higher than the affinity of c-17-1A ($3.0-5.4 \times 10^8$ M$^{-1}$). When the temperature was increased to 37°C no binding of c-17-1A could be observed, whereas m-323/A3 was not affected in its binding.

Competition assays were performed at 4°C, in which [$^{125}$I]m-323/A3 was mixed with WiDr cells preincubated with unlabelled c-17-1A, and [$^{125}$I]c-17-1A was mixed with WiDr cells preincubated with unlabelled m-323/A3 (Figure 1). MAb m-323/A3 completely blocked the binding of c-17-1A to tumour cells, whereas c-17-1A could not inhibit the binding of m-323/A3. Similar results were obtained with the use of OVCAR-3 cells.

*Antigen expression in xenografts*

The antigen distribution in FMa, Ov.Pe and OVCAR-3 xenografts was visualised in an indirect immunoperoxidase assay, in which frozen tumour sections were incubated with m-323/A3, c-17-1A, m-E48 and c-anti-CD4. MAb m-323/A3 stained the membrane and the cytoplasm of all tumour cells in the three xenografts in a moderate to strong manner. MAb c-17-1A also stained the membrane and the cytoplasm of tumour cells, but the intensity of the staining was moderate to weak when compared with that of m-323/A3. The control MAbs m-E48 and c-anti-CD4 did not react with the xenografts.

*Pharmacokinetics and biodistribution*

The blood clearance of m-323/A3 and c-17-1A was investigated in non-tumour-bearing nude mice. No significant differences in the pharmacokinetics between the MAbs could be observed. The initial half-lives of m-323/A3 and c-17-1A were 10.9±4.4 h and 12.5±5.0 h, whereas the terminal half-lives (calculated from 8 to 120 h) were 71.4±13.8 h and 62.7±12.6 h, respectively.

The biodistribution of m-323/A3 and c-17-1A was determined in mice bearing FMa, OVCAR-3 or Ov.Pe xenografts. The retention of both MAbs and their control

**Figure 1** Competition assay of m-323/A3 and c-17-1A with the use of WiDr cells: ○, specific binding of [$^{125}$I]m-323/A3; □, specific binding of [$^{125}$I]c-17-1A; ●, binding of [$^{125}$I]m-323/A3 to cells preincubated with unlabelled c-17-1A; ■, binding of [$^{125}$I]c-17-1A to cells preincubated with unlabelled m-323/A3.

**Table I** Cellular binding characteristics of m-323/A3 and c-17-1A

| *Cell line* | *MAb* | *Temperature (°C)* | *Sites cell$^{-1}$ $\times 10^5$ (±s.d.)* | *$K_a$ (M$^{-1}$) $\times 10^8$ (±s.d.)* |
|---|---|---|---|---|
| OVCAR-3 | m-323/A3 | 4 | 16.7 (±1.4) | 13.0 (±1.0) |
| WiDr | m-323/A3 | 4 | 6.0 (±1.4) | 30.0 (±8.0) |
| OVCAR-3 | c-17-1A | 4 | 10.0 (±2.1) | 3.0 (±0.7) |
| WiDr | c-17-1A | 4 | 5.4 (±1.3) | 5.4 (±0.9) |
| OVCAR-3 | m-323/A3 | 37 | 13.0 (±3.8) | 11.0 (±2.0) |
| WiDr | m-323/A3 | 37 | 11.0 (±2.0) | 13.0 (±3.0) |
| OVCAR-3 | c-17-1A | 37 | No binding | |
| WiDr | c-17-1A | 37 | No binding | |

MAbs m-E48 and c-anti-CD4 in blood and the uptake in tumour tissue and liver are shown in Figure 2. Specific uptake of m-323/A3 was observed in FMa, OVCAR-3 and Ov.Pe xenografts when compared with the control MAb m-E48 ($P<0.01$). MAb c-17-1A localised specifically in the FMa xenograft ($P<0.01$) but the uptake in OVCAR-3 and Ov.Pe xenografts was only slightly higher than that of c-anti-CD4. MAbs m-323/A3 and c-17-1A and their control MAbs localised to a similar extent in the liver. In other normal organs uptake of the MAbs was equal to or lower than the uptake in the liver.

The cumulative absorbed radiation doses delivered by 10 μCi of m-323/A3, c-17-1A, m-E48 and c-anti-CD4 in blood, tumour, liver, spleen and kidney were calculated from the data of the biodistribution experiments (Table II). The absorbed doses in blood exceeded those in the tumours, except for m-323/A3 in mice bearing FMa xenografts. The radiation dose delivered by m-323/A3 to FMa, OVCAR-3 and Ov.Pe xenografts was 4.7-, 2.6- and 2.5-fold, respectively, higher than that delivered by c-17-1A. The liver, spleen and kidney received similar low doses of m-323/A3 and c-17-1A, which were similar to those delivered by m-E48 and c-anti-CD4.

The distribution pattern of m-323/A3 and c-17-1A in FMa and OVCAR-3 xenografts was visualised by autoradiography. MAb m-323/A3 was heterogeneously distributed in FMa

**Figure 2** Percentage of injected dose per gram tissue of iodinated m-323/A3 (○), c-17-1A (□), m-E48 (●) and c-anti-CD4 (■) in blood, tumour and liver of mice bearing FMa, OVCAR-3 or Ov.Pe xenografts. Note: use of a different scale for the uptake of MAbs in FMa tumour tissue.

**Table II** Cumulative absorbed radiation doses in blood, tumours and normal organs of nude mice bearing human ovarian cancer xenografts[a]

| | *Blood* | *Tumour* | *Liver* | *Spleen* | *Kidney* |
|---|---|---|---|---|---|
| FMa | | | | | |
| m-323/A3 | 36 | 165 | 4 | 4 | 5 |
| c-17-1A | 45 | 35 | 8 | 10 | 7 |
| m-E48 | 63 | 15 | 10 | 8 | 10 |
| c-anti-CD4 | 56 | 16 | 11 | 13 | 9 |
| OVCAR-3 | | | | | |
| m-323/A3 | 54 | 31 | 8 | 6 | 9 |
| c-17-1A | 46 | 12 | 5 | 5 | 7 |
| m-E48 | 64 | 10 | 9 | 7 | 11 |
| c-anti-CD4 | 48 | 7 | 6 | 5 | 7 |
| Ov. Pe | | | | | |
| m-323/A3 | 48 | 33 | 7 | 4 | 7 |
| c-17-1A | 44 | 13 | 4 | 4 | 6 |
| m-E48 | 54 | 12 | 7 | 5 | 8 |
| c-anti-CD4 | 49 | 8 | 5 | 5 | 7 |

[a] Absorbed doses were determined from the data of the biodistribution experiments and are expressed in cGy per 10 μCi radiolabelled MAb, calculated over 0–168 h. The % ID $g^{-1}$ in blood at 0 h was derived from the data of the pharmacokinetics in non-tumour-bearing nude mice. The % ID $g^{-1}$ in tumour, liver, spleen and kidney at 0 h was assumed to be 0%.

xenografts, whereas c-17-1A showed a more homogeneous pattern (Figure 3). This phenomenon was observed 24 h after injection and did not change noticeably in the following 3 days. The radioactivity density profiles illustrate the differences in uptake and in distribution between both MAbs. Owing to the lower uptake of m-323/A3 and c-17-1A in OVCAR-3 xenografts, it was more difficult to observe a difference in the distribution between the antibodies, but again m-323/A3 was heterogeneously distributed throughout the tumour (Figure 4).

*Radioimmunotherapy*

In previously performed maximum tolerated dose (MTD) studies the MTD in OVCAR-3 and Ov.Pe xenografts was 400 $\mu$Ci [$^{131}$I]m-323/A3 i.v. given twice with a 2 week interval, whereas in FMa xenografts this was 250 $\mu$Ci. These doses were based on the occurrence of 10–15% weight loss, from which mice had recovered by day 28. Since treatment of FMa xenografts with 100 $\mu$Ci [$^{131}$I]m-323/A3 caused complete tumour regressions, this radiation dose had to be reduced to 50 $\mu$Ci in the treatment experiments.

The tumour growth of control mice and mice treated with [$^{131}$I]m-323/A3 or [$^{131}$I]c-17-1A is shown in Figure 5. When mice were treated with equivalent radiation doses the growth inhibition induced by [$^{131}$I]m-323/A3 and calcualted at day 35 in FMa and Ov.Pe xenografts was better when compared with that of [$^{131}$I]c-17-1A ($P<0.05$, Table III). No difference in growth inhibition was observed in OVCAR-3 xenografts. When corrections were made for the tumour uptake, meaning that a 4.7-fold higher radiation dose of [$^{131}$I]c-17-1A was injected in FMa-bearing mice and a 2.5- and 2.6-fold lower radiation dose of [$^{131}$I]m-323/A3 in Ov.Pe- and OVCAR-3-bearing mice, [$^{131}$I]c-17-1A was more effective in tumour growth inhibition in all three xenografts ($P<0.05$). Especially in FMa xenografts, [$^{131}$I]c-17-1A could induce a growth inhibition of 99% and complete remissions were observed in four of nine tumours. Additional biodistribution experiments with doses of [$^{131}$I]m-323/A3 and [$^{131}$I]c-17-1A adjusted to obtain an equivalent amount of radiation in the tumour confirmed the similarity in absorbed radiation doses (Table III).

Whole-body radiation doses in treated mice were measured up to 4 weeks after the initiation of the treatment. No significant difference in the whole-body clearance was observed between [$^{131}$I]m-323/A3 and [$^{131}$I]c-17-1A in mice bearing FMa or OVCAR-3 xenografts, whereas a slightly faster clearance of [$^{131}$I]c-17-1A was observed in Ov.Pe-bearing mice. The effective whole-body half-life ($\pm$s.e.m.) of [$^{131}$I]m-323/A3 in FMa-, OVCAR-3- and Ov.Pe-bearing mice was $70\pm2.8$ h, $84\pm14$ h and $109\pm8$ h, respectively, whereas this was $99\pm17$ h, $77\pm8.3$ h and $77\pm7$ h, respectively, for [$^{131}$I]c-17-1A. These half-lives were independent of the doses administered. The whole-body absorbed radiation doses were in agreement with the radiation dose administered.

As both m-323/A3 and c-17-1A are able to induce ADCC, the therapeutic effect of unlabelled m-323/A3 and c-17-1A was studied in FMa-bearing mice using the same injection schedule as in the radioimmunotherapy experiments. When mice were treated with m-323/A3 or c-17-1A at protein doses exceeding those used in the radioimmunotherapy experiments (200 $\mu$g) no tumour growth inhibition was induced by either MAb.

### Discussion

In the present study we demonstrated that the high-affinity MAb m-323/A3 targeted better to tumour tissue, but was more heterogeneously distributed when compared with the low-affinity MAb c-17-1A. When mice were treated with equivalent radiation doses of [$^{131}$I]m-323/A3 and [$^{131}$I]c-17-1A, [$^{131}$I]m-323/A3 induced a better growth inhibition in FMa and Ov.Pe xenografts. However, when radiation doses were adjusted to obtain equivalent amounts of radiation in the tumour, [$^{131}$I]c-17-1A induced a better growth inhibition in all three xenografts.

The *in vitro* binding studies demonstrated an important

**Figure 3** Autoradiographs (magnification 5×) and the corresponding radioactivity density profiles of 10 $\mu$Ci [$^{125}$I]m-323/A3 (**a**) and [$^{125}$I]c-17-1A (**b**) in the FMa xenograft 24 h after administration (% ID g$^{-1}$ was 42% for m-323/A3 and 9% for c-17-1A). Note: use of a larger area in (**b**) to obtain a better qualitative activity–density profile.

**Figure 4** Autoradiographs (magnification 4×) and the corresponding radioactivity density profiles of 10 $\mu$Ci [$^{125}$I]m-323/A3 (**a**) and [$^{125}$I]c-17-1A (**b**) in the OVCAR-3 xenograft 24 h after administration (% ID g$^{-1}$ was 11.4% for m-323/A3 and 4.4% for c-17-1A).

role for temperature in the binding capacity of c-17-1A. At 4°C, Scatchard analysis showed a 5-fold higher affinity for m-323/A3 when compared with c-17-1A, whereas the number of binding sites per cell was similar for both MAbs. Similar or even higher affinity ratios at this temperature have been observed by other groups (Pak *et al.*, 1991; Langmuir *et al.*,

**Figure 5** Tumour growth in mice bearing s.c. FMa, OVCAR-3 or Ov.Pe xenografts after i.v. treatment with [$^{131}$I]m-323/A3 or [$^{131}$I]c-17-1A in doses either equivalent in radiation or equivalent in tumour uptake. The arrows show the days of treatment. +, Control; ○, [$^{131}$I]m-323/A3; □, [$^{131}$I]c-17-1A. For doses, see Table III.

**Table III** Growth inhibition (GI), calculated at day 35, induced by $^{131}$I-labelled MAbs in human ovarian cancer xenografts

| | *Equivalent radiation dose* | | | | | | *Equivalent tumour uptake* | | | | | | | |
|---|---|---|---|---|---|---|---|---|---|---|---|---|---|---|
| | *[$^{131}$I]m-323/A3* | | | *[$^{131}$I]c-17-1A* | | | *[$^{131}$I]m-323/A3* | | | | *[$^{131}$I]c-17-1A* | | | |
| | *μCi* | *μg* | *GI (%)* | *μCi* | *μg* | *GI (%)* | *μCi* | *μg* | *cGy*[a] | *GI (%)* | *μCi* | *μg* | *cGy*[a] | *GI (%)* |
| FMa | 50 | 15 | 93[b] | 70 | 20 | 46 | 50 | 15 | 533 | 93 | 240 | 67 | 474 | 99[c] |
| OVCAR-3 | 300 | 85 | 93 | 400 | 116 | 92 | 150 | 42 | 312 | 74 | 400 | 116 | 380 | 92[c] |
| Ov.Pe | 285 | 81 | 67[b] | 400 | 116 | 51 | 160 | 46 | 395 | 32 | 400 | 116 | 441 | 51[c] |

[a] Cumulative absorbed radiation doses expressed in cGy by multiplying the integrated μCi h $g^{-1}$ by the g cGy $μCi^{-1}$ $h^{-1}$ factor for $^{131}$I calculated over 0–96 h. [b] Significantly different from an equivalent radiation dose of [$^{131}$I]c-17-1A ($P < 0.05$). [c] Significantly different from a dose of [$^{131}$I]m-323/A3, resulting in a similar amount of radiation in the tumour ($P < 0.05$).

1992; Oredipe *et al.*, 1992). At 37°C we observed no binding of c-17-1A, whereas the binding of m-323/A3 was not affected. Lower binding of 17-1A with increasing temperature has been reported before (Oredipe *et al.*, 1992; Langmuir *et al.*, 1992). Changes in membrane fluidity at 37°C resulting in a physical barrier for 17-1A to bind to its epitope could be the cause of the reduced binding of 17-1A, as was suggested by the group of Langmuir. *In vivo*, however, we could still demonstrate specific uptake of c-17-1A in human ovarian cancer xenografts.

Competition assays at 4°C showed a complete inhibition of the binding of c-17-1A to tumour cells by m-323/A3, whereas m-323/A3 was still able to bind to tumour cells that were preincubated with c-17-1A. This phenomenon has been reported before (Pak *et al.*, 1991; Langmuir *et al.*, 1992) and can be explained by the different affinities. In addition, Langmuir *et al.* (1992) suggested that the binding site for c-17-1A may be masked when m-323/A3 is bound to its epitope, whereas the occupation of the binding site for c-17-1A does not hamper the binding of m-323/A3.

In comparison with c-17-1A, m-323/A3 showed a more intense staining pattern in frozen sections of all three human ovarian cancer xenografts. The stronger reactivity of 323/A3 may be due to its higher affinity, but other factors could also explain the difference in staining intensity. First, the epitope for 17-1A is known to be sensitive for fixatives (Herlyn *et al.*, 1986), which may result in a decrease in binding sites for c-17-1A during the processing for immunohistochemistry. Second, a different glycosylated form of the antigen may be expressed in some tumour types, which can be recognised by m-323/A3 but not by c-17-1A (Velders *et al.*, 1994). In addition, Thampoe *et al.* (1988) have described two forms of the antigen, a single chain of 38 kDa with a disulphide-linked intramolecular loop, and a derived disulphide-linked dimer consisting of a 32 kDa and a 6 kDa subunit. Both forms may be recognised by m-323/A3 but not by c-17-1A.

MAb m-323/A3 targeted better to tumour tissue than c-17-1A. The cumulative absorbed radiation doses in FMa, OVCAR-3 and Ov.Pe xenografts delivered by m-323/A3 were higher than those delivered by c-17-1A, even when corrections were made for the immunoreactive fractions of the MAbs after radiolabelling. Also, m-323/A3 remained longer in tumour tissue than c-17-1A. These results can most likely be explained by the extreme difference in affinity between both MAbs at 37°C. However, other factors such as differences in the quantity and accessibility of the epitopes could have an effect on the tumour uptake as well. Autoradiography showed a heterogeneous distribution pattern of a low protein dose of m-323/A3 in tumour tissue, whereas c-17-1A was more homogeneously distributed. These findings are in agreement with the hypothesis of a perivascular binding for high-affinity MAbs and a deeper penetration for low-affinity MAbs in tumour tissue (Juweid *et al.*, 1992).

Although m-323/A3 showed a similar strong reactivity pattern with frozen sections of all three human ovarian cancer xenografts *in vitro*, the tumour uptake of m-323/A3 *in vivo* varied between the xenografts. Blumenthal *et al.* (1992*a*) have also found a variation in uptake of a radiolabelled anti-CEA MAb in different human colon cancer xenografts, which was most likely due to differences in the intratumoral and intracellular distribution of CEA. We found m-323/A3 staining of the membrane and the cytoplasm of tumour cells throughout the whole xenograft tissue sections, indicating a homogeneous distribution of the antigen. To obtain more insight into the differences in the uptake of m-323/A3 in these xenografts, we are currently analysing other factors of influence, including tumour perfusion, tumour vascularisation pattern and the antigen content.

In our treatment experiments mice were injected with doses of [$^{131}$I]c-17-1A and [$^{131}$I]m-323/A3, which were adjusted to be equivalent in radiation or equivalent in tumour uptake. MAb [$^{131}$I]m-323/A3 was superior in growth inhibition in two xenografts when compared with an equivalent radiation dose of [$^{131}$I]c-17-1A. This is most likely a result of the higher uptake of m-323/A3 in these xenografts. When similar absorbed radiation doses in the tumours were obtained a superiority in growth inhibition was observed for [$^{131}$I]c-17-1A in all three xenografts. We expect the deeper tumour penetration of c-17-1A resulting in a more homogeneous distribution of radiation as the most likely explanation for the better efficacy of [$^{131}$I]c-17-1A. Although the whole-body absorbed radiation dose was higher in mice treated with [$^{131}$I]c-17-1A, this was not expected to have a therapeutic effect on the s.c. tumours. In small animals there is virtually no self-absorption of $\gamma$-rays (Wahl, 1994). Therefore, tumour growth inhibition induced by $^{131}$I-labelled MAbs can mostly be attributed to tumour-absorbed $\beta$-particles, which have a mean range of 0.8 mm (Jurcic and Scheinberg, 1994).

The effect of MAb affinity on the efficacy of radioimmunotherapy has been studied before, but the preference for low- or high-affinity MAbs was still elusive. *In vivo*, better tumour-targeting properties have been reported for a high affinity anti-CEA MAb (Hansen *et al.*, 1993), but the MAb was equally effective in radioimmunotherapy when compared with a similar radiation dose of a low-affinity anti-CEA MAb (Blumenthal *et al.*, 1992*b*). In this study no corrections were made for the immunoreactivity of the MAbs after radiolabelling. Other *in vivo* studies have reported an improved localisation as well as a therapeutic advantage for radiolabelled high-affinity MAbs against the TAG-72 antigen when compared with a low affinity anti-TAG-27 MAb (Colcher *et al.*, 1988; Schlom *et al.*, 1992). Even with a 2.5- to 3-fold lower radiation dose of the high-affinity MAbs, which might compensate for the lower amount of absorbed radiation dose delivered by the low-affinity MAb, better anti-tumour effects were observed for the high-affinity MAbs. This is in contrast with the present experiments, in which we clearly show that the low-affinity MAb c-17-1A is superior to the high-affinity MAb m-323/A3 in tumour growth inhibition when similar amounts of radiation are delivered to the tumour. Nevertheless, 323/A3, preferably in the chimeric form, may be favourable for use in radioimmunotherapy as higher radiation doses of c-17-1A required to obtain similar or better anti-tumour effects will be associated with increased side-effects. In addition, m-323/A3 targets better to tumour tissue and may therefore be more efficient in immunoscintigraphy of tumour lesions in patients.

**Acknowledgements**
This work was supported by the Dutch Cancer Society, Amsterdam, The Netherlands.

### References

BLUMENTHAL RD, SHARKEY RM, KASHI R, NATALE AM AND GOLDENBERG DM. (1992*a*). Physiological factors influencing radioantibody uptake: a study of four human colonic carcinomas. *Int. J. Cancer*, **51,** 935–941.

BLUMENTHAL RD, SHARKEY RM, HAYWOOD L, NATALE AM, WONG GY, SIEGEL JA, KENNEL SJ AND GOLDENBERG DM. (1992*b*). Targeted therapy of athymic mice bearing GW-39 human colonic cancer micrometastases with $^{131}$I-labeled monoclonal antibodies. *Cancer Res.*, **52,** 6036–6044.

BUCHSBAUM DJ, BRUBAKER PG, HANNA DE, GLATFELTER AA, TERRY VH, GUILBAULT DM AND STEPLEWSKI Z. (1990). Comparative binding and preclinical localization and therapy studies with radiolabeled human chimeric and murine 17-1A monoclonal antibodies. *Cancer Res.*, **50** (suppl.3), 993s–999s.

CHATAL JF, SACCAVINI JC, FUMOLEAU P, DOUILLARD JY, CURTET C, KREMER M, LE MEVEL B AND KOPROWSKI H. (1984). Immunoscintigraphy of colon carcinoma. *J. Nucl. Med.*, **25,** 307–314.

CHEN TR, DRABKOWSKI D, HAY RJ, MACY M AND PETERSON W. (1987). WiDr is a derivative of another colon adenocarcinoma cell line, HT-29. *Cancer Genet. Cytogenet.*, **27**, 125–134.

COLCHER D, MINELLI MF, ROSELLI M, MURARO R, SIMPSON-MILENIC D AND SCHLOM J. (1988). Radioimmunolocalization of human carcinoma xenografts with B72.3 second generation monoclonal antibodies. *Cancer Res.*, **48**, 4597–4603.

DILLMAN LT. (1969). Radionuclide decay schemes and nuclear parameters for use in radiation-dose estimation. MIRD pamphlet no.4. *J. Nucl. Med.* suppl.2, 6–13.

EDWARDS DP, GRZYB KT, DRESSLER LG, MANSEL RE, ZAVA DT, SLEDGE GW AND McGUIRE WL. (1986). Monoclonal antibody identification and characterization of a Mr 43,000 membrane glycoprotein associated with human breast cancer. *Cancer Res.*, **46**, 1306–1317.

FOLEY GE, LAZARUS H, FARBER S, UZMAN BG, BOONE BA AND McCARTHY RE. (1965). Continuous culture of human lymphoblasts from peripheral blood of a child with acute leukemia. *Cancer*, **18**, 522–529.

HAISMA HJ, HILGERS J AND ZURAWSKI VR. (1986). Iodination of monoclonal antibodies for diagnosis and radiotherapy using a convenient one vial method. *J. Nucl. Med.*, **27**, 1890–1895.

HAMILTON TC, YOUNG RC, McKOY WM, GROTZINGER KR, GREEN JA, CHU EW, WHANG-PENG J, ROGAN AM, GREEN WR AND OZOLS RF. (1983). Characterization of a human ovarian carcinoma cell line (NIH:OVCAR-3) with androgen and estrogen receptors. *Cancer Res.*, **43**, 5379–5389.

HANSEN HJ, GOLDENBERG DM, NEWMAN ES, GREBENAU R AND SHARKEY RM. (1993). Characterization of second-generation monoclonal antibodies against carcinoembryonic antigen. *Cancer*, **71**, 3478–3485.

HERLYN M, STEPLEWSKI Z, HERLYN D AND KOPROWSKI H. (1986). CO 17-1A and related monoclonal antibodies: their production and characterization. *Hybridoma*, **5** (suppl.1), S3–S10.

JURCIC JG AND SCHEINBERG DA. (1994). Recent developments in the radioimmunotherapy of cancer. *Curr. Opin. Immunol.*, **6**, 715–721.

JUWEID M, NEUMANN R, PAIK C, PEREZ-BACETE MJ, SATO J, VAN OSDOL W AND WEINSTEIN JN. (1992). Micropharmacology of monoclonal antibodies in solid tumors: direct experimental evidence for a binding-site barrier. *Cancer Res.*, **52**, 5144–5153.

KOPROWSKI H, STEPLEWSKI Z, MITCHELL K, HERLYN M, HERLYN D AND FUHRER P. (1979). Colorectal carcinoma antigens detected by hybridoma antibodies. *Somat. Cell. Genet.*, **5**, 957–972.

LANGMUIR VK, MENDONCA HL AND WOO DV. (1992). Comparisons between two monoclonal antibodies that bind to the same antigen but have differing affinities: uptake kinetics and $^{125}I$-antibody therapy efficacy in multicell spheroids. *Cancer Res.*, **52**, 4728–4734.

LINDMO T, BOVEN E, CUTTITTA F, FEDORKO J AND BUNN PA. (1984). Determination of the immunoreactive fraction of radiolabeled monoclonal antibodies by linear extrapolation to binding at infinite antigen excess. *J. Immunol. Methods*, **72**, 77–89.

LINNENBACH AJ, SENG BA, WU S, ROBBINS S, SCOLLON M, PYRC JJ, DRUCK T AND HUEBNER K. (1993). Retroposition in a family of carcinoma-associated antigen genes. *Mol. Cell. Biol.*, **13**, 1507–1515.

MELLSTEDT H, FRÖDIN JE, MASUCCI G, RAGNHAMMAR P, FAGERBERG J, HJELM AL, SHETYE J, WERSÄLL P AND ÖSTERBORG A. (1991). The therapeutic use of monoclonal antibodies in colorectal carcinoma. *Semin. Oncol.*, **18**, 462–477.

MEREDITH RF, LOBUGLIO AR, PLOTT WE, ORR RA, BREZOVICH IA, RUSSELL CD, HARVEY EB, YESTER MV, WAGNER AJ, SPENCER SA, WHEELER RH, SALEH MN, ROGERS KJ, POLANSKY A, SALTER MM AND KHAZAELI MB. (1991). Pharmacokinetics, immune response, and biodistribution of iodine-131-labeled chimeric mouse/human IgG1,k 17-1A monoclonal antibody. *J. Nucl. Med.*, **32**, 1162–1168.

MOLTHOFF CFM, CALAME JJ, PINEDO HM AND BOVEN E. (1991). Human ovarian cancer xenografts in nude mice: Characterization and analysis of antigen expression. *Int. J. Cancer*, **47**, 72–79.

MOMBURG F, MOLDENHAUER G, HAMMERLING GJ AND MOLLER P. (1987). Immunohistochemical study of the expression of a $M_r$ 34,000 human epithelium-specific surface glycoprotein in normal and malignant tissues. *Cancer Res.*, **47**, 2883–2891.

OREDIPE OA, BARTH RF, ROTARU JH AND STEPLEWSKI Z. (1992). Modulation of monoclonal antibody affinity and antigenic receptor site expression on human colon cancer cells. *Antibody Immunoconj. Radiopharm.*, **5**, 295–306.

PAK KY, NEDELMAN MA, FOGLER WE, TAM SH, WILSON E, VAN HAARLEM LJ, COLOGNOLA R, WARNAAR SO AND DADDONA PE. (1991). Evaluation of the 323/A3 monoclonal antibody and the use of technetium-99m-labeled 323/A3 Fab′ for the detection of pan adenocarcinoma. *Nucl. Med. Biol.*, **18**, 483–497.

QUAK JJ, BALM AJM, BRAKKEE JGP, SCHEPER RJ, HAISMA HJ, BRAAKHUIS BJM, MEIJER CJLM AND SNOW GB. (1989). Localization and imaging of radiolabelled monoclonal antibody against sqamous-cell carcinoma of the head and neck in tumor-bearing nude mice. *Int. J. Cancer*, **44**, 534–538.

RIETHMÜLLER G, SCHNEIDER-GÄDICKE E, SCHLIMOK G, SCHMIEGEL W, RAAB R, HÖFFKEN K, GRUBER R, PICHLMAIER H, HIRCHE H, PICHLMAYR R, BUGGISCH P AND WITTE J. (1984). Randomised trial of monoclonal antibody for adjuvant therapy of resected Dukes' C colorectal carcinoma. *Lancet*, **343**, 1177–1183.

SCHLOM J, EGGENSPERGER D, COLCHER D, MOLINOLO A, HOUCHENS D, MILLER LS, HINKLE G AND SILER K. (1992). Therapeutic advantage of high-affinity anticarcinoma radioimmunoconjugates. *Cancer Res.*, **52**, 1067–1072.

THAMPOE IJ, NG JS AND LLOYD KO. (1988). Biochemical analysis of a human epithelial surface antigen: differential cell expression and processing. *Arch. Biochem. Biophys.*, **267**, 342–352.

VELDERS MP, LITVINOV SV, WARNAAR SO, GORTER A, FLEUREN GJ, ZURAWSKI VR AND CONEY LR. (1994). New chimeric anti-pancarcinoma monoclonal antibody with superior cytotoxicity-mediating potency. *Cancer Res.*, **54**, 1753–1759.

WAHL RL. (1994). Experimental radioimmunotherapy. *Cancer*, **73**, 989–992.